# Assessment of fractal dimension changes in periapical trabecular patterns following single-visit and multi-visit nonsurgical endodontic treatment with different medicaments: a retrospective study

**DOI:** 10.2340/aos.v84.45201

**Published:** 2025-12-22

**Authors:** Hikmet Kaan Kirci, Emrah Karataslioglu

**Affiliations:** Department of Endodontics, Faculty of Dentistry, University of İzmir Kâtip Çelebi, İzmir, Turkey

**Keywords:** Dental radiography, fractal, root canal medicaments, retrospective studies, root canal therapies

## Abstract

**Objective:**

This study aimed to compare time-dependent changes in fractal dimension (FD) and periapical index (PAI) values of periapical trabecular bone in the lesion areas following single- and multi-visit endodontic treatments using different intracanal medicaments.

**Material and Methods:**

Data from 62 mandibular molars treated between March and December 2023 were analyzed and assigned to three groups: Group 1, multi-visit treatment with calcium hydroxide [Ca(OH)₂] (*n* = 20); Group 2, multi-visit treatment with chlorhexidine (CHX) gel (*n* = 21); and Group 3, single-visit treatment without medicament (*n* = 21). Follow-up data were collected until December 2024. Three periapical radiographs, baseline, 6-months, and 12 months after treatment, were evaluated. Fractal analysis was performed on a region of interest (ROI) near the infected root apex, and periapical healing was also assessed using PAI scores. PAI scores were compared using the Kruskal–Wallis test; FD values and their correlation with PAI were analyzed by two-way analysis of variance (ANOVA) and Spearman’s test. Categorical variables were compared using the chi-square and Fisher–Freeman–Halton tests.

**Results:**

PAI scores significantly decreased in all groups at 6 and 12 months compared with baseline (*p* < 0.001), with no intergroup differences (*p* > 0.05). FD values significantly increased over time in all groups (*p* < 0.001). The magnitude of FD change and gender-related differences were not significant (*p* > 0.05). No correlation was observed between PAI and FD values (*p* > 0.05).

**Conclusions:**

Single- and multi-visit endodontic treatments resulted in similar periapical healing outcomes. The type of intracanal medicament did not influence FD or PAI values. Fractal analysis is a valuable, noninvasive tool for assessing periapical bone healing over time.

## Introduction

During root canal treatment, chemomechanical cleaning and shaping can effectively remove bacteria and their by-products from the root canal system. In the case of a necrotic tooth with radiological changes in the periradicular area, persistent microorganisms in the anatomical complexities may increase the risk of treatment failure. Therefore, additional disinfection should be applied with an interappointment intracanal medication [1].

Calcium hydroxide (Ca(OH)₂) is the frequently used intracanal medicament in endodontics due to its many benefits, including its high alkalinity, tissue dissolution capacity, endotoxin neutralization ability, and antibacterial qualities [2]. In addition, it controls inflammatory processes and induces repair procedures [3]. Chlorhexidine (CHX), the other commonly used intracanal medicament, is a broad-spectrum antibacterial agent that has been highly promoted due to its retentive nature, low toxicity, and ability to effectively combat strains that are resistant to Ca(OH)₂ [4].

Over the last 30 years, advances in methods and technologies such as microscopic magnification, heat-resistant nickel-titanium instruments, and new irrigation protocols have improved the effectiveness and efficiency of endodontic treatment overall, including both single- and multi-visit approaches. These innovations have enabled clinicians to manage complex cases more predictably and, in many situations, more efficiently in a single visit [5]. Clinical studies have shown that there is no significant difference between one visit and two visits in the resolution of endodontically induced periapical lesions [6–9].

Different radiographic methods such as periapical radiography, panoramic radiography, and cone beam computed tomography (CBCT) are used to evaluate the health of periapical tissues after root canal treatment. Although CBCT has advantages such as allowing 3-dimensional imaging of dental structures, its routine use in the follow-up of root canal treatment is limited due to its higher cost and exposure to ionizing radiation compared to 2-dimensional radiographs [10]. Periapical radiography is frequently utilized instead of panoramic radiography because it uses less ionizing radiation and permits a more thorough assessment of the area of interest [11]. In clinical and epidemiologic studies, a variety of indices (namely, the periapical index (PAI), Strindberg index, and probability index) are used to assess the status of periapical tissues by the evaluation of periapical radiographs [12]. PAI is a radiographic interpretation method used to evaluate periapical healing, initially defined by Ørstavik et al. in 1986. Its validity relies on the use of reference radiographs of teeth that have been histologically diagnosed. The index employs an ordinal scale with five scores, ranging from ‘healthy’ to ‘severe periodontitis with exacerbating features’ [13]. However, a major drawback of this assessment method is its subjectivity, as substantial intra- and interobserver variations may negatively affect the reliability and reproducibility of studies [14].

Fractal analysis, first introduced by Mandelbrot in 1967, is a mathematical and morphological image processing method used to determine the fractal dimension (FD) of trabecular bone through radiographic imaging. This method provides a quantitative and objective assessment of bone microarchitecture, offering valuable insights into the mechanisms underlying bone regeneration. It has been widely used to assess root canal treatments’ success in teeth with apical lesions and can be applied to two-dimensional radiographs [15]. One of the significant advantages of fractal analysis is its resilience to minor variations in projection angle and radiodensity, ensuring reliable and reproducible measurements in routine intraoral radiographic examinations, and the data required for calculation of this parameter can be obtained without the need for invasive procedures [14, 16].

After conducting a comprehensive literature review, we found no previous studies that used fractal analysis to evaluate radiographic changes in periapical trabecular patterns, following single-visit nonsurgical endodontic treatment and multi-visit nonsurgical endodontic treatment with different intracanal medicaments. The aim of this study was to evaluate and compare time-dependent changes in FD and PAI values of periapical lesions using periapical radiographs at baseline (immediately after treatment) and at 6- and 12-month follow-ups. These changes were assessed following single-visit and multi-visit nonsurgical root canal treatments performed with different intracanal medicaments. The null hypothesis was that there would be no significant differences in FD and PAI changes between single-visit and multi-visit treatments, nor between the intracanal medicaments [Ca(OH)₂] and (CHX).

## Materials and methods

### Case selection and sample size calculation

In this retrospective study, the data have been obtained from clinical studies conducted by Prof. Dr. Emrah Karataslioglu and from the clinical records of patients whose treatments have been monitoring. A total of 62 mandibular molars treated between March 2023 and December 2023 were included. The retrospective data collection was carried out after obtaining approval from the Izmir Katip Çelebi University Non-Interventional Clinical Research Ethics Committee (No: 0435/2023). The principles specified in the 1975 Helsinki Declaration, updated in 2013, were followed in the conduct of this investigation. A written-informed consent was obtained from each patient. Follow-up data were collected until December 2024.

This study included samples with the following criteria: (1) periapical radiograph images of patients, taken immediately after treatment, 6-months, and 12 months posttreatment follow-ups; (2) patients aged between 18 and 65 years and who received endodontic treatment with asymptomatic apical periodontitis with a score of 3 or more on the PAI scoring system; (3) data from systemically and periodontally healthy patients who had not used antibiotics, anti-inflammatory painkillers, systemic corticosteroids, and other drugs that might affect bone metabolism in the last 6 months; (4) periapical radiograph images of treated mandibular molars (#36, 37, 46, and 47) with no artifacts, superimpositions, or image distortions, from which the relevant area could be monitored clearly. Images of teeth with vertical root fractures, external/internal resorption, and surgical endodontic treatment were excluded from the study.

The G*Power program, version 3.1.5 (Franz Faul, Chris tian-Albrechts Universität Kiel, Kiel, Germany), was used to calculate the sample size. For the calculation, the mean and standard deviation values of FD at baseline and 12-month follow-up were used, taking into account the previously published study by Tosun et al., similar to our study [17]. According to power analysis software, this study required a minimum of 20 participants for each group with a power of 80%, effect size of 0.25, and α = 0.05. Therefore, the data of 62 treated lower molars of 42 patients were included in our study. Only one tooth in 28 patients, two teeth in eight patients, and three teeth in six patients were evaluated in this study.

Study groups were as follows:

- Group 1: Multi-visit group with Ca(OH)₂ as an intracanal medicament, (*n* = 20).
- Group 2: Multi-visit group with CHX gel as an intracanal medicament (*n* = 21).
- Group 3: Single-visit group with no intracanal medicament (*n* = 21).

### Treatment protocol

For all patients, 1.5 mL of anesthetic solution (4% articaine with 1:200.000 epinephrine) was administered to provide anesthesia. Proper rubber dam isolation was applied to the relevant teeth, and access cavities were prepared according to standard guidelines. The canal orifices were identified using a loupe (Univet, Ontario, Canada) and irrigated with 2.5% NaOCl (Chloraxid 2.5% [w/v]; PPH Cerkamed, Stalowa Wola, Poland). Working lengths were determined with an electronic apex locator (Woodpex III, Guilin Woodpecker Medical Instrument, Guangxi, China) and recorded. Root canal shaping was performed using a rotary endodontic file system (Protaper Universal, Dentsply Maillefer, Ballaigues, Switzerland). Mechanical shaping procedures were carried out using shaping files (SX, S1, and S2) and then finished by finishing files (F1, F2, F3, F4, and F5) to match the pre-determined apical constriction size. Throughout mechanical preparation, canals were copiously irrigated with 2.5% NaOCl.

For single-visit treatments, final irrigation was performed with 2 mL of 2.5% NaOCl per canal using a side-vented irrigation needle (Brokers Confort, Medical Brokers Adam Cieslak, Lodz, Poland). Ultrasonic activation (UltraX, Eighteeth, Jiangsu, China) was performed for 20 s, followed by rinsing with distilled water. Subsequently, 17% ethylenediaminetetraacetic acid (EDTA) (Imicryl, Konya, Turkey) was applied for 1 min, rinsed with distilled water, and a final 1-min irrigation with 2.5% NaOCl was conducted. Canals were dried using paper points (Diadent Group International INC., South Korea), and obturation was completed using gutta-percha and a resin-based sealer (Ad-Seal, Meta Biomed, Chungcheongbuk-do, South Korea) via the cold lateral compaction technique. The access cavity was cleaned with alcohol-soaked cotton, sealed with glass ionomer cement (Meron, Voco, Cuxhaven, Germany), and referred to the Restorative Dentistry Department for definitive restorations. Postoperative instructions were provided, and patients were scheduled for follow-up at 6 and 12 months.

For multi-visit treatments, intracanal medication was performed with Ca(OH)₂ (Imicryl, Konya, Turkey) or 2% CHX gel (Consepsis Scrub, Ultradent, South Jordan, Utah, USA) for 10 days. Medicament was applied to each canal with a sterile 25# K-file, set 1 mm shorter than the working length. The pulp chamber was closed with teflon tape and sealed with glass ionomer cement (Meron, Voco, Cuxhaven, Germany) as a temporary filling material. At the second visit, rubber dam isolation was reapplied. Temporary restorative material and teflon tape were removed. Canals were irrigated with 17% EDTA. The working length was reconfirmed under NaOCl irrigation. Intracanal medicament was removed using an ultrasonic device and Master Apical File. Final irrigation and obturation protocol was performed in the same manner as a single-visit group. Postoperative instructions were provided, and patients were scheduled for follow-up at 6 and 12 months.

### Radiography

All digital periapical radiographs of posterior mandibular regions were obtained using the same periapical radiographic X-ray device, Myray RXAC (CEFLA Dental Group Via Bicocca IMOLA BO, Italy). All images were taken in the same parallel technique and the same radiographic exposure settings (65 kVp, 7 mA, and 0.32 s exposure time) to ensure image standardization. D-speed intraoral photostimulated phosphorus plate (#2) (PSPIX^®^ Imaging Plates, Sopro, France) was used with a posterior teeth film holder (Endo-Bite, Kerr), which allowed the vertical angle of the X-ray to reach both the long axis of the tooth and the image receptor at 90°. The images were then scanned with the same plate scanner (Dürr Vista Scan Mini View) and recorded in high-resolution JPEG format.

Periapical radiographs were selected based on the inclusion criteria from those taken immediately after treatment as well as 6- and 12-month follow-ups. To minimize researcher bias, the radiographs were randomized using a free randomization tool (www.randomizer.org). Following the randomization process, the researchers were blinded to the group allocation of each radiograph, ensuring that they were unaware of which study group each sample belonged to.

### Fractal analysis

**Figure 1:**
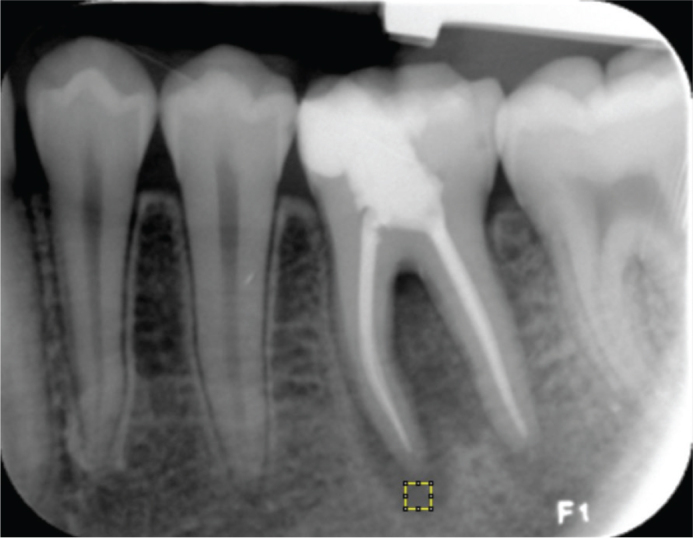
Determination of the ROI in a periapical radiograph. The yellow square shows the ROI.

**Figure 2:**
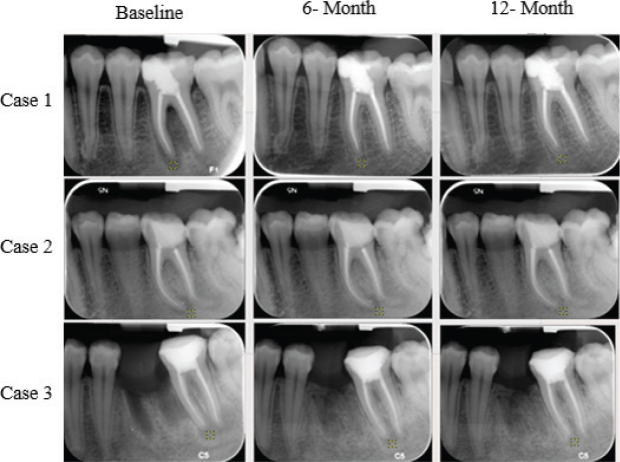
ROI selection for three different cases in periapical radiographs.

**Figure 3:**
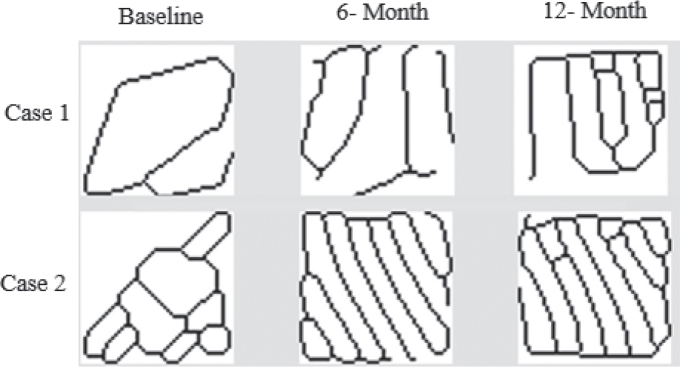
Skeletonized images of ROI from baseline, 6-month, and 12-month radiographs of two different cases.

Fractal analysis was performed using the method developed by White & Rudolph [18]. Image J version 1.3 software (National Institutes of Health, Bethesda, MD, USA) was used to analyze images. Initially, regions of interest (ROIs) were selected from the periapical lesion area and positioned 1 mm away from the root apex in the radiographs taken immediately after treatment for each patient. The ROI was repositioned from the same location based on this distance in follow-up (6- and 12-month) radiographs (Figure 1). Each ROI chosen in the three radiographs had the same size (50 × 50 pixels) and location for every tooth. The chosen ROI was therefore repeatable and standardized (Figure 2). Each selected 50 × 50 pixels ROI was clipped and duplicated. The duplicated image was blurred by applying a “Gaussian Blur” (sigma = 35 pixels). This blurring process left only significant density variations in the image after removing high and moderate details representing the bone’s varying thickness and superficial soft tissue covering. The blurred image was subtracted from the original image, and 128 gray tones were added for each pixel position to separate the bone marrow from the trabecular structure. Then, the image was converted to an 8-bit format using the “Type” option, and each image was converted to a two-color (black-and-white) image using the “Threshold” option; these borders represented the trabecular structure and the bone marrow contours. Image noise was minimized using the “Erode” option, followed by expansion of the available areas using the “Dilate” option to make them more prominent. By using the “Invert” option, the trabecular bone’s contours were visible as white parts in the image turned black and black areas turned white. The trabecular structure’s contours were identified by lines using the “Skeletonize” option, which also prepared the structure for fractal analysis. The “Fractal box count” option in the “Analyze” tab was then used to calculate the FD (Figure 3).

Fractal analyses were performed by a single researcher. To test intra-observer reliability, 50% of the images were reanalyzed 1 month later after the first one by the same researcher. The correlation coefficient for intra-observer reliability was calculated (0.97).

### PAI score analysis

Periapical status was also evaluated with the PAI. The periapical tissues of each patient were radiographically assessed using the PAI 5-step scoring system on each of three radiographs (immediately after treatment, 6-month, and 12-month posttreatment follow-ups) as follows:

PAI 1: Normal periapical structure.

PAI 2: Minor changes in the bone structure, not pathognomonic of apical periodontitis.

PAI 3: Structural bone changes with mineral loss, characteristic of apical periodontitis.

PAI 4: Well-defined apical radiolucency, characteristic of apical periodontitis.

PAI 5: Severe periodontitis with exacerbation and bone expansion [13].

Radiographs were analyzed by two experienced endodontist observers independent of the study. First, to calibrate the observers, 100 radiographs, independent of the study, were evaluated by the observers beforehand and discussed to reach a consensus. Then, the radiographs belonging to the study were numbered and asked to be evaluated without informing the observers which group they belonged to. Each observer evaluated the radiographs twice with an interval of 1 month. If there was a discrepancy between the observers during the evaluation, re-scoring and consensus were tried to be achieved. When the discrepancy was repeated, the higher score was taken into consideration. Correlation coefficients were calculated for interobserver and intra-observer reliability. These were determined and recorded as 0.81 and 0.89, respectively.

### Statistical analysis

Statistical analyses were performed by using IBM SPSS Statistics Standard Concurrent User V 29 (IBM Corp., Armonk, New York, USA) statistical package program. Descriptive statistics were given as a number of units (*n*), percentage (%), mean ± standard deviation, median, minimum, and maximum values. The normal distribution of the numerical variables was evaluated by the Shapiro Wilk normality test. Variance homogeneity of the groups was analyzed by the Levene’s test. Since the PAI scores were not normally distributed, comparisons between groups were made by Kruskal-Wallis analysis. Repeated measures two-way analysis of variance (ANOVA) was used to compare the baseline, 6, and 12-month FD values according to the groups. The relationships between PAI scores and FD values were evaluated with Spearman correlation coefficient. The Pearson chi-square and Fisher-Freeman-Halton exact tests were used to compare the groups with categorical variables. Two-way ANOVA was used to compare the relationship between gender and change in fractal dimension (ΔFD) both within and between groups. Statistically significance was considered as *P* < 0.05.

## Results

Table 1 presents the descriptive characteristics of patients according to the groups. There were no significant differences among the three groups in terms of age or sex distribution (*p* > 0.05). The FD values increased significantly in both the multi-visit and single-visit groups following root canal treatment at all time intervals (*p* < 0.05). However, inter-group comparisons revealed no significant difference in FD values between groups at any measurement time (*p* > 0.05) (Table 2). PAI scores significantly reduced following root canal treatment in all three groups. Six- and 12-month scores were statistically lower than immediately after treatment (baseline) values (*p* < 0.05); however, the difference between the 6- and 12-month scores was not statistically significant (*p* > 0.05) (Table 3). As shown in Table 4, there was no significant difference in the magnitude of FD change among study groups across all time intervals (*p* > 0.05). Similarly, no significant differences were observed between females and males regarding the time-dependent changes in FD values in both intra-group and inter-group comparisons (*p* > 0.05). Although an inverse relationship was observed between PAI scores and FD values — indicating that as one increased, the other tended to decrease at all measurement times; this correlation was not statistically significant at baseline (*r* = −0.164, *p* > 0.05), 6 months (*r* = −0.244, *p* > 0.05), and 12 months (*r* = −0.040, *p* > 0.05).

**Table 1:** Descriptive characteristics of patients according to the groups.

	Groups			*P*-value
	Ca(OH)₂ *n* = 15	CHX *n* = 13	Single visit *n* = 14	
**Age (years)** (mean ± SD)	30.8 ± 13.6	40.9 ± 13.6	33.9 ± 13.5	0.181^†^
**Gender** *n* (%)				0.931^‡^
Male	8 (53.3)	6 (46.2)	7 (50)	
Female	7 (46.7)	7 (53.8)	7 (50)	

SD: standard deviation; *n*: Number of patients, %: Column percentage, Age is presented as *mean ± standard deviation*.

† : One-way analysis of variance (*ANOVA*),

‡ : Pearson chi-square test.

**Table 2:** Comparison of FD values according to the groups.

	Groups			*P*-value
	CaOH *n* = 21	ChX *n* = 20	Single visit *n* = 21	
Immediately after treatment (baseline)	1.36 ± 0.11^a^	1.36 ± 0.13^a^	1.35 ± 0.13^a^	0.957^δ^
6-month	1.42 ± 0.12^b^	1.45 ± 0.12^b^	1.45 ± 0.10^b^	0.699^δ^
12-month	1.51 ± 0.09^c^	1.49 ± 0.10^c^	1.52 ± 0.08^c^	0.575^δ^
*p*-value	< 0.001 ^δ^	< 0.001 ^δ^	< 0.001 ^δ^	

FD values are given as mean ± standard deviation. δ: Two-way analysis of variance in repeated measures, superscripts a, b, and c indicate the difference between within-group measurement times. Measurements with the same superscript are not statistically different.

**Table 3:** Comparison of PAI values according to the groups.

	Groups			*P*-value
	CaOH *n* = 21	ChX *n* = 20	Single visit *n* = 21	
Immediately after treatment (baseline)	3 (3–5)^a^	3 (3–5)^a^	4 (3–5)^a^	0.338^&^
6-month	2 (1–3)^b^	2 (1–3)^b^	2 (1–4)^b^	0.661^&^
12-month	1 (1–2)^b^	1 (1–2)^b^	1 (1–4)^b^	0.884^&^
*P*-value	< 0.001^¥^	< 0.001^¥^	< 0.001^¥^	

PAI values are summarized as median (min-max). &: Kruskal-Wallis test, ¥: Friedman analysis, superscripts a and b indicate the difference between within-group measurement times. Measurements with the same superscript are not statistically different.

**Table 4:** Comparison of time-intervals changes in FD values between groups.

	Groups			*P*-value
	Ca(OH)₂ *n* = 21	CHX *n* = 20	Single Visit *n* = 21	
6 month – Baseline	0.065 ± 0.050	0.091 ± 0.096	0.100 ± 0.096	0.360^†^
12 month – 6 month	0.093 ± 0.097	0.046 ± 0.045	0.076 ± 0.085	0.178^†^
12 month – Baseline	0.157 ± 0.105	0.137 ± 0.104	0.177 ± 0.123	0.525^†^

Changes in FD values are given as mean ± standard deviation.

† : One-way analysis of variance.

## Discussion

The assessment of root canal treatment outcomes relies on the analysis of radiographic findings in conjunction with the presence or absence of clinical signs and symptoms [15]. This retrospective study is the first work in the literature to use fractal analysis of periapical radiographs to assess FD changes in apical lesion regions following single- and multi-visit root canal treatment. Additionally, it evaluates the healing process of primary multi-visit root canal treatments performed with different intracanal medicaments in cases of apical periodontitis.

The results of this study revealed a significant increase in the FD values following root canal treatment over time in all groups. The observed increase in FD values reflects the reorganization of the trabecular structure during periapical tissue healing. Previous studies have similarly shown that FD values increase with healing, indicating new bone formation and the restructuring of periapical tissues [15, 16]. Furthermore, the observed significant reduction in PAI scores across all groups supports the FD values in terms of the effectiveness of root canal treatment in promoting periapical healing. However, the absence of a significant difference in FD changes between groups suggests that the choice of intracanal medicament (Ca(OH)₂ vs. CHX) and treatment regimens (single vs. multi) may not have a substantial impact on the fractal characteristics and trabecular structure of the periapical bone during periapical tissue healing. Thus, the null hypothesis was accepted.

The absence of a statistically significant difference between single-visit and multi-visit treatment groups aligns with previous research [7, 19, 20]. A clinical study by Penesis et al. also reported similar success rates for single- and multi-visit endodontic treatments [8]. Additionally, this finding is further supported by other studies, which found no significant difference in the clinical outcomes between these two treatment regimens [21, 22]. However, several studies have reported conflicting results, indicating that multi-visit treatments may provide superior clinical outcomes during periapical tissue healing compared to single-visit. In a previous study, it was found that multi-visit protocols could lead to better long-term success, particularly in cases of complex or infected root canal systems [23]. On the other hand, a study by Sathorn et al. reported that a single-visit root canal treatment was slightly more effective than multi-visit; the healing rate was 6.3% higher in treated cases, but the difference was not statistically significant [6]. These conflict results may be attributed to differences in study designs, treatment protocols, or patient characteristics. So, several factors such as the anatomical complexity of the root canal system, the ability to obtain infection control, procedural complications, and preoperative diagnosis may play an important role in the decision-making process of 1- versus 2-visit endodontics. Besides these objective factors, subjective factors like patients’ signs and symptoms and individual patient characteristics may explain the variation in results across different studies, necessitating further long-term studies [5, 7, 24].

When evaluating the efficacy of intracanal medicaments, the results of this study demonstrate that there is no significant difference in the change of FD values between the two intracanal medicaments (Ca(OH)₂ and CHX). This finding indicates that both agents exhibit similar efficacy concerning the apical healing process and contribute similarly to the reorganization of the trabecular structure during periapical healing. Ca(OH)₂ is often preferred due to its alkaline nature and antibacterial effects, which assist in tissue healing [2]. In contrast, CHX has been reported to enhance antibacterial efficacy, particularly against bacteria resistant to Ca(OH)₂, providing an advantage in specific cases [4]. However, this study found no conclusive evidence, suggesting that one intracanal medicament offers superior antibacterial efficacy or clinical benefits over the other. The findings of the current study align with previous research [19]. Nevertheless, other studies comparing the antimicrobial efficacy of CHX and Ca(OH)₂ as intracanal medicaments suggest that CHX demonstrates superior effectiveness against a broad spectrum of bacteria [24, 25]. This highlights a potential variation in efficacy depending on the complexity of the root canal system and the nature of the infection, which may explain the mixed results in the literature. These findings also imply that the fundamental principles of endodontic treatment, including effective disinfection, proper chemomechanical preparation, and obturation, maybe more critical to healing than the specific intracanal medicament used.

PAI is a widely accepted radiographic index that serves as a reliable indicator of endodontic success and correlates with histological findings [13, 25]. However, it is a subjective evaluation method with interobserver variability, which may affect its reliability and may limit its ability to fully reflect the biological complexity of periapical healing [14]. In contrast, fractal analysis is a quantitative, noninvasive, and objective measure that reflects changes in bone density [14, 26], mineral loss, and architecture [18, 27]. Lower FD indicates higher porosity, while higher FD suggests a denser and more complex bone structure [28]. Another advantage is its ability to detect early periapical bone changes following root canal treatment. A previous study has shown an increase in FD in radiographs as early as 3 months posttreatment [15]. Moreover, it remains unaffected by minor variations in projection geometry or radiodensity [29], making it highly effective and sensitive in identifying subtle trabecular bone changes in various conditions, including periodontal disease, post-surgical situations, and systemic disorders [26, 30, 31].

Despite being a powerful tool for quantitatively assessing bone structural changes, fractal analysis has certain limitations. While some studies suggest that FD remains unaffected by minor variations in projection, such as film contrast, tube angulation, and irradiation in images [32], others reported that parameters such as kVp, mAs, and X-ray angulation can influence FD values to some extent [18, 33]. To minimize the impact of these potential inconsistencies, the same periapical radiography device with identical radiographic exposure settings was used. Additionally, all images were taken using the same parallel technique. Another limitation of fractal analysis is its susceptibility to biological variability. Trabecular bone structure is influenced not only by bone density but also by processes such as demineralization, new bone formation, and trabecular remodeling. For instance, in the demineralization process, although bone density decreases, the increased visibility of trabecular patterns may lead to higher FD values. On the contrary, the connection of large trabecular struts can reduce trabecular density, resulting in a decrease in FD [31]. This variability limits FA’s use as a stand-alone diagnostic tool and emphasizes the necessity of supplementary radiographic and clinical evaluations to ensure accurate interpretation. Therefore, in this study, periapical conditions were also evaluated using PAI to determine whether the changes in FD values after root canal treatment were due to newly formed trabecular patterns or decalcification. The significant increase in FD values was attributed to increased trabeculation as part of the healing process. This result was also supported by PAI scores that significantly decreased over time.

There is no consensus on whether ROI selection is an effective factor in the outcomes of fractal analysis. Conflicting reports have been reported in the literature. Shrout et al. reported that FD is affected by ROI size, shape, and location [34]. On the contrary, Amer et al. have reported that there is no significant effect of ROI selection on the outcomes of fractal analysis [35]. In the present study, to avoid this limitation, the ROI was placed in the same size and position in all radiographs. For each tooth, the position and size of the ROI were kept the same in all three radiographs (50x50 pixels). Thus, the ROI was standardized and reproducible. Furthermore, the ROI was chosen to exclude surrounding anatomical structures such as the tooth root and mandibular canal. Therefore, only changes in the lesion area were evaluated. However, we acknowledge that this choice represents a localized area of the alveolar bone and therefore may not fully reflect the overall bone architecture of the jaw.

The results of the present study revealed a negative correlation between PAI and FD, along with a decrease in PAI scores, and a significant increase in FD after root canal treatment. In other words, the change in FD values was supported by PAI scores in the present research. Our results were in agreement with previous studies [17, 26, 36]. However, the lack of statistical significance in the correlation may be attributed to differences in the technical methodologies used for periapical healing assessment. While PAI is a radiographic scoring system based on periapical radiographs, providing a standardized method to assess and classify periapical status in research and clinical contexts [13], FD offers a more detailed structural mathematical analysis of trabecular bone modifications. Future studies integrating both methods in larger sample sizes, possibly in conjunction with advanced imaging techniques such as CBCT, may further elucidate their respective roles and enhance diagnostic accuracy in endodontic follow-up evaluations.

As an additional finding of our study, there was no significant difference between the genders in terms of time-related increases in FD values. In accordance with the results of our study, several studies reported no significant difference in periapical healing between males and females after a 1-year follow-up from endodontic treatment [17, 26, 30, 36]. However, conflicting results were reported by Marquis et al. [37] that the success of endodontic treatment was higher in females than in males; in contrast, Smith et al. [38] reported a higher rate of success in men. The discrepancies in these results can be explained by differences in population, age range, healing assessment method, follow-up time, and sample size.

This study has certain limitations. As a retrospective study, standardization challenges in data collection and evaluation may have occurred. Additionally, the follow-up period was limited to 12 months, preventing a comprehensive assessment of long-term effects. Although all root canal treatments were performed by a single operator, which could introduce operator-related bias, this also ensured procedural standardization and consistency across all cases. Moreover, the use of two-dimensional periapical radiographs may not fully represent the three-dimensional nature of periapical healing. Future studies with longer follow-up periods, larger patient groups, and advanced imaging techniques such as CBCT could provide a more extensive evaluation of periapical healing.

## Conclusions

Within the limitations of this study, both single- and multi-visit root canal treatments resulted in comparable periapical healing, as reflected by significant reductions in PAI scores and increases in FD values over time. The type of intracanal medicament, including CHX gel and calcium hydroxide [Ca(OH)₂], did not significantly influence these parameters. Fractal analysis proved to be a reliable and quantitative method for assessing periapical bone healing. Further studies with larger sample sizes and longer follow-up periods are recommended to validate and extend these findings.
